# Weekday Affects Attendance Rate for Medical Appointments: Large-Scale Data Analysis and Implications

**DOI:** 10.1371/journal.pone.0051365

**Published:** 2012-12-13

**Authors:** David A. Ellis, Rob Jenkins

**Affiliations:** School of Psychology, University of Glasgow, Glasgow, United Kingdom; University of Warwick, United Kingdom

## Abstract

The financial cost of missed appointments is so great that even a small percentage reduction in Did Not Attend (DNA) rate could save significant sums of money. Previous studies have identified many factors that predict DNA rate, including patient age, gender, and transport options. However, it is not obvious how healthcare providers can use this information to improve attendance, as such factors are not under their control. One factor that is under administrative control is appointment scheduling. Here we asked whether DNA rate could be reduced by altering scheduling policy. In Study 1, we examined attendance records for 4,538,294 outpatient hospital appointments across Scotland between January 1st 2008 and December 31st 2010. DNA rate was highest for Mondays (11%), lowest for Fridays (9.7%), and decreased monotonically over the week (Monday-Friday comparison [χ^2^(1, N  = 1,585,545)  = 722.33, p<0.0001]; Relative Risk Reduction 11.8%). This weekly decline was present for male and female patient groups of all ages, but was steeper for younger age groups. In Study 2, we examined attendance records for 10,895 appointments at a single GP clinic in Glasgow. Here again, DNA rate was highest for Mondays (6.2%), lowest for Fridays (4.2%), and decreased monotonically over the week (Monday-Friday comparison [χ^2^(1, N  = 4767)  = 9.20, p<0.01]; Relative Risk Reduction 32.3%). In two very different settings, appointments at the beginning of the week were more likely to be missed than appointments at the end of the week. We suggest that DNA rate could be significantly reduced by preferentially loading appointments onto high-attendance days.

## Introduction

The Did Not Attend (DNA) rate for outpatient appointments at UK hospitals is approximately 12% [1], in line with other nations [2]. These missed appointments cost the UK health system an estimated £600 M per year [3]. This figure is so large that even small reductions in DNA rate could save significant sums. For example, cutting non-attendance by a tenth (from 12% to 10.8%) would cut the annual cost by a tenth (from £600 M to £540 M). In other words, an absolute reduction in DNA rate of just 1.2% could save £60 M per year. Improving attendance would also reduce health risk to patients [4]–[6].

Despite longstanding interest in the causes of missed appointments, practical recommendations for reducing their prevalence are scarce. This is partly because the causes of non-attendance are not well understood. Previous studies have often reported conflicting results [7]–[10], possibly due to their reliance on small samples from disparate settings [4 5 11–13]. Nevertheless, there is some consensus on a number of determinants [14]. These include patient factors such as age [7 9 15], gender [11 16 17], and transport logistics [8 11 18], as well as clinic or practitioner factors such as booking efficiency [5 19–22] and staff-patient rapport [21 23 24]. Common to many of these factors is that they do not suggest any straightforward intervention strategies. The age, gender, and transport options of patients are outside of the healthcare provider’s control; and changes in institutional practice can be costly to implement due to additional equipment or procedural requirements (e.g. telephone reminders [25]).

Here we propose that Did Not Attend (DNA) rates could be cheaply reduced by modifying appointment allocation strategy. Recent psychological studies have shown that different days of the week evoke distinct emotional responses [26]. Monday is most negative, Friday is most positive, and emotional tone brightens steadily over the intervening days. Given that attending a medical appointment may place additional burdens on the patient (e.g. organising work absence or travel logistics; confronting unpleasant treatment [14]), we hypothesised that the contrasting emotional characters of different weekdays might translate to contrasting attendance rates. Specifically, we expected DNA rate follow the psychological peaks and troughs of the weekly cycle, with emotionally positive days boosting patient resilience and improving attendance [27].

To test this hypothesis we conducted two independent analyses, the first based on national outpatient data across Scottish hospitals, and the second based on attendance data from a general practitioners’ (GP) clinic in Glasgow, Scotland. To anticipate, we found the predicted pattern in both data sets: DNA rates were significantly higher on Mondays than on Fridays, and showed a graded decrease over the week. The data imply that millions of pounds could be saved by preferentially loading appointments onto high attendance days.

### Ethics Statement

All patient data used in our studies was anonymous. For Study 1, our request for dates of missed and attended appointments cleared Information Services Division Scotland ethical procedures, and the dataset was delivered in anonymous form. IRB approval was not required for the study.

## Study 1: National Outpatient Data

### Analysis

Information Services Division Scotland provided a complete record of outpatient hospital appointments for the whole of Scotland, from January 1st 2008 to December 31st 2010 (4,538,294 appointments in total). To our knowledge, this is the largest corpus of data for which DNA rates have been analysed. Weekend appointments were infrequent (<2% of cases) and were excluded from the analysis. To test for a weekly trend in non-attendance, we categorised the remaining 4,463,369 appointments according to weekday, and calculated DNA rates (i.e. the percentage of appointments that were missed) separately for each weekday.

### Results

Table 1 shows DNA rate as a function of weekday. As can be seen from the table, DNA rate is highest on Mondays (11%), lowest on Fridays (9.7%), and declines monotonically through the week (Relative Risk Reduction 11.8%). Chi-square comparison of the frequency of attended versus non-attended appointments revealed a highly reliable difference between Monday and Friday [χ^2^(1, N  = 1,585,545)  = 722.33, p<0.0001].

**Table 1:** Overall DNA rates by weekday in Study 1.

DNA rate					Weekday effect	
Monday	Tuesday	Wednesday	Thursday	Friday	Mon-Fri	Mon-Fri
n = 967912	n = 1032417	n = 957447	n = 887960	n = 617633	difference	RRR*
11.0%	10.9%	10.3%	10.1%	9.7%	1.3%	**11.8%**

* Relative Risk Reduction.

Table 2 shows DNA rates in Study 1 separately for male and female patients and for different age bands. The overall demographic trends are consistent with those in previous studies. DNA rates were generally higher for males than for females, and highest overall for young males [14 16 28]. Importantly, the decline in DNA rate over the week is seen in both male and female patients, and in all age bands. Note however that the steepness of decline varies with patient age. Specifically, weekday predicts DNA rate more strongly in younger patients than in older patients.

**Table 2:** DNA rates in Study 1.

			DNA rate (Male patients)										Weekday effect		
			Mon		Tue		Wed		Thu		Fri		Mon-Fri	Mon-Fri	
Age band			n = 396567		n = 433157		n = 394641		n = 359344		n = 261418		difference	RRR*	
0–9	n = 150538		12.4%		11.0%		11.7%		10.8%		10.9%		1.5%	**12.1%**	
10–19	n = 141121		13.7%		13.9%		13.5%		13.1%		13.0%		0.7%	**5.1%**	
20–29	n = 179614		24.0%		23.9%		23.2%		22.7%		22.1%		1.9%	**7.9%**	
30–39	n = 201834		20.9%		20.8%		19.4%		19.5%		18.6%		2.3%	**11.0%**	
40–49	n = 260940		15.0%		15.1%		14.4%		14.6%		13.9%		1.1%	**7.3%**	
50–59	n = 267595		10.1%		10.1%		10.0%		9.6%		9.4%		0.7%	**6.9%**	
60–69	n = 286983		6.5%		6.3%		6.1%		6.1%		5.7%		0.8%	**12.3%**	
70–79	n = 239210		5.4%		5.0%		5.0%		5.0%		4.6%		0.8%	**14.8%**	
80+	n = 117292		6.5%		5.8%		5.8%		5.8%		5.0%		1.5%	**23.1%**	
All ages	n = 1845127		12.5%		12.3%		11.7%		11.6%		11.1%		1.4%	**11.2%**	
				**DNA rate (Female patients)**									**Weekday effect**		
				**Mon**		**Tue**		**Wed**		**Thu**		**Fri**	**Mon-Fri**		**Mon-Fri**
**Age Band**				**n = 571345**		**n = 599260**		**n = 562806**		**n = 528616**		**n = 356215**	**difference**		**RRR** *
0–9		n = 117160		12.4%		11.5%		12.0%		10.4%		10.7%	1.7%		**13.7%**
10–19		n = 159731		13.0%		12.4%		12.0%		11.8%		11.8%	1.2%		**9.2%**
20–29		n = 354468		16.1%		16.0%		14.1%		14.0%		13.8%	2.3%		**14.3%**
30–39		n = 375747		12.4%		12.5%		11.5%		11.5%		10.9%	1.5%		**12.1%**
40–49		n = 412726		10.9%		10.6%		10.2%		10.1%		9.5%	1.4%		**12.8%**
50–59		n = 365421		8.1%		8.1%		7.5%		7.4%		7.4%	0.7%		**8.6%**
60–69		n = 335763		5.4%		5.5%		5.2%		5.1%		4.7%	0.7%		**13.0%**
70–79		n = 302603		5.4%		5.4%		5.1%		4.8%		4.7%	0.7%		**13.0%**
80+		n = 194623		7.1%		6.3%		6.3%		6.3%		5.8%	1.3%		**18.3%**
All ages		n = 2618242		10.0%		9.8%		9.2%		9.0%		8.7%	1.3%		**13.0%**

* Relative Risk Reduction.

Percentage non-attendance rates for each weekday are shown separately for male patients (top) and female patients (bottom) in different age bands.

To establish the generality of the basic effect, and to examine its relevance for scheduling policy in individual clinics, we next conducted a similar weekday analysis based on data from a single general practice.

## Study 2: Single Practice Data

### Analysis

A General Practitioners’ (GP) clinic in Glasgow, Scotland made available a complete anonymous record of their appointment scheduling for January 1st 2009 to December 31st 2009 (10,895 appointments in total). Note that the practice in question did not normally schedule GP appointments for Tuesdays or weekends, and these days were excluded from the analysis. Percentage DNA rates for the remaining weekdays were calculated as in Study 1.

### Results

As can be seen from Table 3, the weekday pattern is strikingly similar to that seen in Study 1. Overall DNA rate was again highest for Mondays (6.2%), lowest for Fridays (4.2%), and decreased over the intervening days (Relative Risk Reduction 32.3%). Chi-square analysis to compare the frequency of attended versus non-attended appointments confirmed that the difference between Monday and Friday was statistically highly robust [χ^2^(1, N  = 4767)  = 9.20, p<0.01].

**Table 3:** DNA rates by weekday in Study 2.

DNA rate					Weekday effect	
Monday	Tuesday	Wednesday	Thursday	Friday	Mon-Fri	Mon-Fri
n = 2511	N/A	n = 2856	n = 3272	n = 2256	difference	RRR*
6.2%	N/A	5.9%	4.6%	4.2%	2.0%	**32.3%**

* Relative Risk Reduction.

## Discussion

In two large datasets, we found that DNA rates for medical appointments declined monotonically over the week. This pattern was present for both male and female patients and in all age groups, but was stronger in younger age groups. Importantly, it also generalised across national hospital and single practice settings.

Although the present study does not address the causes of the weekday effect directly, we note that the pattern seen here in real world attendance rates echoes the pattern seen in emotional responses to weekday cues [27]. In line with our predictions, attendance was systematically higher on days that elicit emotionally positive associations (e.g. Friday), and lower on days that elicit emotionally negative associations (e.g. Monday). These findings raise the possibility that medical appointments may be harder to face on some weekdays than on others. This interpretation chimes with the many psychological reasons that patients give for non-attendance (e.g. fear of bad news [24], fear of unpleasant treatment [24], negative relationships with staff [23 25]). A complete explanation will require further study. For now, the data clearly show that appointments at the beginning of the week were missed more often than those at the end of the week. This observation suggests a simple strategy for reducing non-attendance: where practicable, load appointments towards the end of a week. Interestingly, the actual loading pattern in the hospital data (but not the single practice data) was opposite to this. That is, appointments were disproportionately loaded to the beginning of a week, where DNA rates are highest (see Table 2). The contrast between ideal and actual scheduling patterns underscores the practical value of our analysis: Significant health and financial savings could be achieved simply by changing the distribution of appointments over the week.

We note that the effect of weekday on DNA rate in Study 1 is numerically smaller than the effect of patient gender (overall Monday-Friday difference 1.3%, Relative Risk Reduction 11.8%; overall Male-Female difference 2.5%, Relative Risk Reduction 20.8%). Importantly however, appointment allocation policy can be changed easily, whereas the gender of patients can not. For this reason, we suggest that the weekday effect is of greater practical significance despite its smaller size.

For several reasons, we believe that the effect of weekday on DNA rates should be of interest to practitioners, managers, and politicians alike. First, it points to clear recommendations that can be easily acted upon, as outlined above. Second, it brings attendance problems under the control of the service provider, rather than relying on education or punishment of the patient [15 17]. Third, changing appointment allocation policy should be inexpensive compared with other approaches to reducing DNA rate, as it does not require any additional equipment or procedures (cf. telephone or text reminders [29 30]). Finally, reductions in DNA rate achieved through reminder schemes and through scheduling improvements may be additive, if these very different approaches target separable causes of non-attendance (e.g., forgetting and motivation, respectively).

A straightforward trial of the weekday effect in a clinical setting could involve shifting appointments from Mondays to Fridays, and comparing overall attendance rates before and after this intervention. We would very interested to hear of any such trials. For now, we show that weekday predicts likelihood of attendance for medical appointments. Exploiting this weekday effect could save money and improve patient care.
